# Maf1 Is a Novel Target of PTEN and PI3K Signaling That Negatively Regulates Oncogenesis and Lipid Metabolism

**DOI:** 10.1371/journal.pgen.1004789

**Published:** 2014-12-11

**Authors:** Beth M. Palian, Aarti D. Rohira, Sandra A. S. Johnson, Lina He, Ni Zheng, Louis Dubeau, Bangyan L. Stiles, Deborah L. Johnson

**Affiliations:** 1 Department of Biochemistry and Molecular Biology, Keck School of Medicine, University of Southern California, and the Norris Comprehensive Cancer Center, Los Angeles, California, United States of America; 2 Department of Pharmaceutical Sciences, School of Pharmacy, Keck School of Medicine, University of Southern California, and the Norris Comprehensive Cancer Center, Los Angeles, California, United States of America; 3 Department of Pathology, Keck School of Medicine, University of Southern California, and the Norris Comprehensive Cancer Center, Los Angeles, California, United States of America; Stanford University Medical Center, United States of America

## Abstract

Maf1 was initially identified as a transcriptional repressor of RNA pol III-transcribed genes, yet little is known about its other potential target genes or its biological function. Here, we show that Maf1 is a key downstream target of PTEN that drives both its tumor suppressor and metabolic functions. Maf1 expression is diminished with loss of PTEN in both mouse models and human cancers. Consistent with its role as a tumor suppressor, Maf1 reduces anchorage-independent growth and tumor formation in mice. PTEN-mediated changes in Maf1 expression are mediated by PTEN acting on PI3K/AKT/FoxO1 signaling, revealing a new pathway that regulates RNA pol III-dependent genes. This regulatory event is biologically relevant as diet-induced PI3K activation reduces Maf1 expression in mouse liver. We further identify lipogenic enzymes as a new class of Maf1-regulated genes whereby Maf1 occupancy at the FASN promoter opposes SREBP1c-mediated transcription activation. Consistent with these findings, Maf1 inhibits intracellular lipid accumulation and increasing Maf1 expression in mouse liver abrogates diet-mediated induction of lipogenic enzymes and triglycerides. Together, these results establish a new biological role for Maf1 as a downstream effector of PTEN/PI3K signaling and reveal that Maf1 is a key element by which this pathway co-regulates lipid metabolism and oncogenesis.

## Author Summary

Obesity is a strong risk factor for human cancers, yet the biological basis for this is unclear. In addition to aberrant growth, abnormal lipid synthesis is a hallmark of cancer cells. Our results have identified a novel role for Maf1 in suppressing both lipid biogenesis and tumor formation. Maf1 elicits these biological responses through its ability to repress genes that that synthesize lipids and regulate biosynthetic capacity. Maf1 amounts are regulated through a critical cellular pathway involving PTEN/PI3K/Akt/FoxO1, which is deregulated in many human cancers. Our results support the idea that deregulation of this pathway in cancer cells results in decreases in cellular Maf1, resulting in both abnormal growth and lipid synthesis. Thus, Maf1 represents a novel link between lipid metabolism and oncogenic transformation providing a new molecular basis for the strong association between obesity and cancer.

## Introduction

Obesity, characterized by an elevation in circulating lipids, has been associated with increased incidence of cancer [1]. A hallmark of cancer cells is an increase in *de novo* fatty acid synthesis. This occurs as a result of induction in the expression and function of lipogenic enzymes including fatty acid synthase (FASN) and acetyl-CoA carboxylase (ACC) [2], [3]. Elevated expression of these lipogenic enzymes is observed in the early premalignant stage of a variety of cancers and is thought to play a critical role in tumorigenesis and cancer progression. Enhanced expression of these genes is correlated with poor survival. Consistent with these findings, inhibiting expression of these genes in cancer cell lines promotes growth arrest and apoptosis, prompting efforts to design therapeutic agents to target FASN [4]. However, the side effects of existing FASN inhibitors have so far precluded their clinical use in cancer. Thus, a better understanding of how lipid homeostasis is regulated will provide important new therapeutic opportunities.

PTEN is a critical tumor suppressor and is the second most frequently mutated gene in human cancer, behind only p53 [5]. Loss of PTEN results in the activation of the PI3 kinase (PI3K) signaling pathway, which plays a pivotal role in the proliferation and survival of cancer cells. PI3K/AKT signaling induces *de novo* lipogenesis through enhanced expression and activation of the transcription factor sterol regulatory element-binding protein-1 (SREBP1) [6]. Insulin treatment or constitutive AKT activation promotes the nuclear accumulation of SREBP1 and its recruitment to the lipogenic enzyme gene promoters, resulting in their coordinate induction. That SREBP activates lipogenic genes is well established; whether there are processes that can oppose SREPB-mediated activation is not known.

Maf1 is a transcriptional repressor initially characterized in *S. cerevisiae* where it is a central node for the repression of RNA polymerase (pol) III-dependent gene expression [7]. Maf1 inhibits transcription by its direct recruitment to tRNA and 5S rRNA gene promoters through its interaction with RNA pol III [8]. In contrast to its role in yeast, in humans, Maf1 negatively regulates transcription resulting from all three nuclear RNA polymerases [9]. In addition to its function in repressing RNA pol III-dependent transcription, Maf1 directly represses RNA pol II-dependent transcription of the central transcription initiation factor, TATA-binding protein (TBP). As TBP is can be a limiting component for RNA pol I-dependent rRNA synthesis, Maf1-mediated regulation of TBP expression also modulates rRNA gene transcription. Cellular levels of TBP determine the proliferative [10] and transforming properties of cells [11]. Elevated synthesis of tRNAs and rRNAs, a hallmark of neoplasia, is required for cellular transformation and tumorigenesis [12], [13]. The ability of Maf1 to repress the transcription of genes encoding TBP, tRNAs and rRNAs supports the idea that it plays a key role in suppressing oncogenesis. Consistent with this concept, expression of Maf1 in PTEN-deficient human glioblastoma cells inhibits anchorage-independent growth [9].

To explore the biological role of Maf1 and to investigate how it is regulated, we asked whether the tumor suppressor PTEN modulates Maf1 expression. *Pten*-deficient mouse liver and prostate tissues exhibited marked decreases in Maf1 protein, whereas re-expression of PTEN in human PTEN-deficient cells increased Maf1 expression. Consistent with these results, a decrease in nuclear Maf1 expression is observed in both human hepatocellular carcinoma and prostate cancers that have lost PTEN. Furthermore, increased Maf1 expression represses cellular transformation. PTEN represses Maf1 expression through its ability to regulate the PI3K/AKT2/FoxO1 signaling pathway. Consistent with this idea, diet-induced insulin signaling reduces Maf1 expression in mouse liver. Changes in FoxO1 signaling alter Maf1-targeted RNA pol III-dependent gene transcription, defining a new pathway for the regulation of these genes. In addition, we identify a new class of Maf1-regulated genes. Maf1 negatively regulates the expression of the lipogenic enzymes, FASN and ACC1. Maf1 occupies the FASN promoter to oppose SREBP1c-mediated activation. Consistent with these findings, decreased Maf1 expression in human hepatoma cells promotes the accumulation of intracellular lipids whereas overexpression of Maf1 in mouse liver inhibits diet-induced lipogenesis. Together these studies identify a new mechanism for Maf1 regulation through PTEN/PI3K signaling and a new target of PTEN that plays an important role in the ability of this pathway to negatively regulate both tumorigenesis and lipid metabolism.

## Results

### PTEN positively regulates Maf1 expression

**Figure 1:**
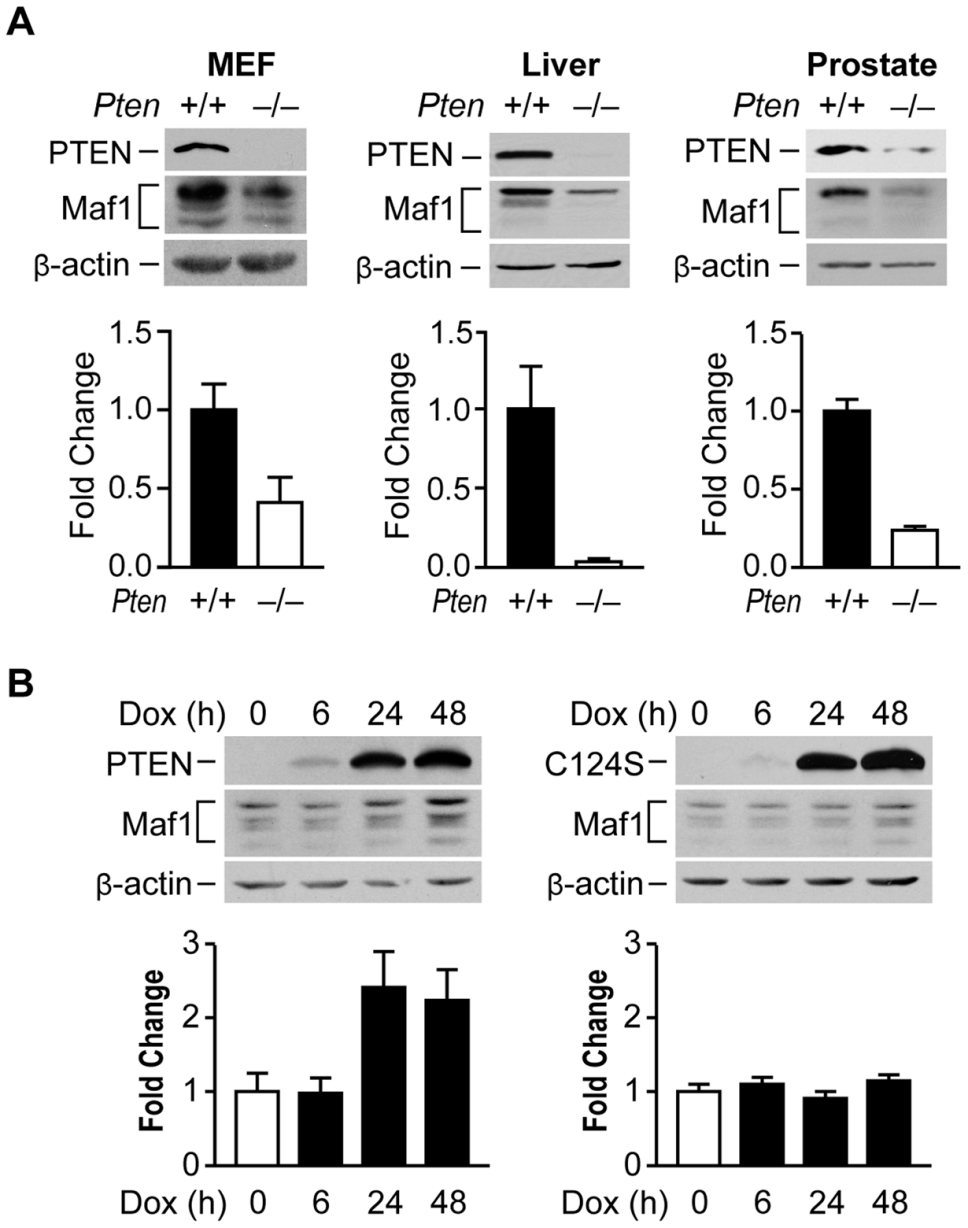
PTEN regulates Maf1 protein expression. (**A**) Maf1 expression is decreased in mouse cells and tissues lacking PTEN. Protein lysates were isolated from wild-type and *Pten*−/− MEFs; livers of 1 month old *Pten^loxP/loxP^; Alb-Cre*− (+/+; n = 4) and *Pten^loxP/loxP^*; *Alb-Cre+* (−/−; n = 3) mice; and prostates of 5.2 week old *Pten^loxP/loxP^; PB-Cre+* mice (+/+; n = 3) and *Pten^loxP/loxP^*; *PB-Cre*− littermate controls (−/−; n = 3). Lysates were subjected to immunoblot analysis with antibodies against the proteins indicated. Densitometry analysis revealed statistically significant changes in Maf1 expression (Student t-test, MEF *p* = 0.0426; liver *p* = 0.0097; prostate *p* = 0.0046) (**B**) Induction of wild type PTEN, but not a phosphatase defective mutant form induces Maf1 expression in cells lacking endogenous PTEN. U87 cells engineered to stably express inducible PTEN or phosphatase defective PTEN-C124S were used. Protein lysates were isolated from cells treated with 1 µg/ml doxycycline at times indicated. Immunoblot analysis was performed using antibodies as indicated. Densitometry analysis revealed significant differences between no doxycycline control and 24 and 48 hr doxycycline treatment (ANOVA, *p*<0.0001). Maf1 amounts were normalized to β-actin. The graphs represent quantification of 3 independent experiments. Values shown are the means ±S.E.

To begin to explore how Maf1 might be regulated, we asked whether the tumor suppressor, PTEN, might control Maf1 levels. In MEFs, loss of *Pten* resulted in a substantial decrease in Maf1 protein levels (Fig. 1A). In tissue lysates derived from mouse models where *Pten* is selectively deleted in the liver or prostate, Maf1 expression was similarly decreased (Fig. 1A). The reduction in Maf1 is observed before the onset of tumors in these tissues [14], [15] indicating that the changes in Maf1 are concurrent with the loss of *Pten*. Consistent with a reduction in Maf1 upon loss of PTEN, induction of PTEN expression in PTEN-deficient human glioblastoma U87 cells resulted in an increase in Maf1 expression and required the phosphatase activity of PTEN (Fig. 1B). Together, these results demonstrate that PTEN regulates Maf1 expression in a variety of cell types.

### Maf1 is deregulated in human cancer and suppresses cellular transformation

**Figure 2:**
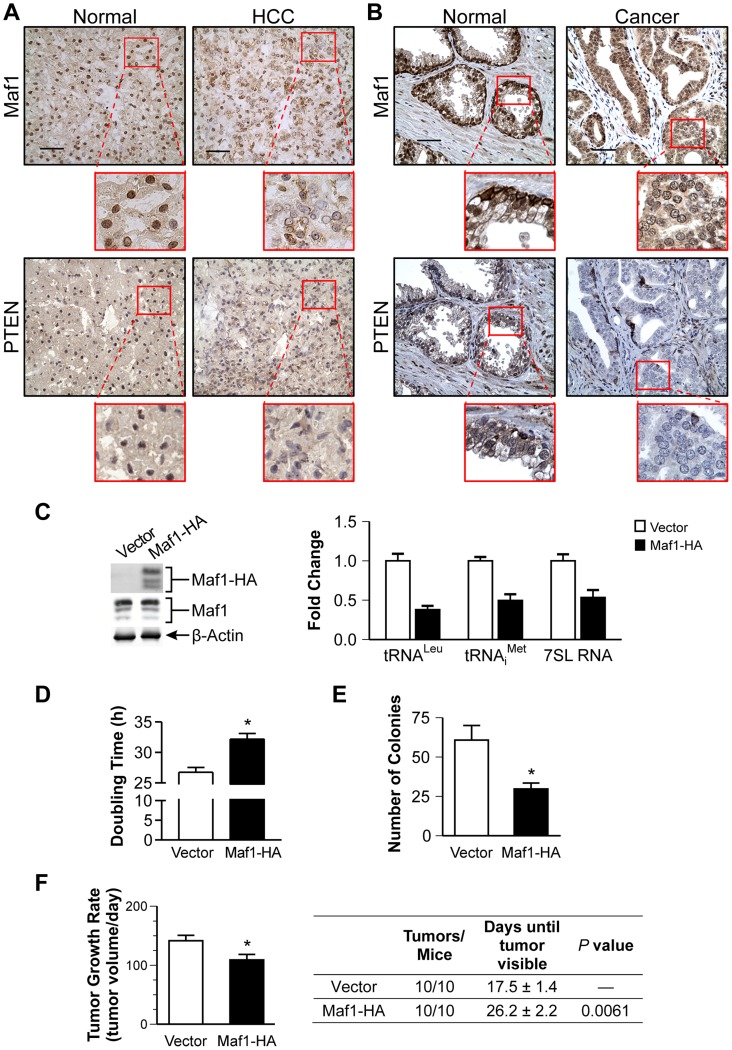
Increased Maf1 expression suppresses cellular transformation and tumorigenesis, consistent with its diminished expression in human cancer tissues. Nuclear Maf1 expression is decreased in PTEN negative human prostate and liver cancers. Immunohistochemistry of frozen human liver tissue (**A**) and paraffin-embedded human prostate tissue (**B**) with Maf1 or PTEN antibodies. Photomicrographs show representative staining of cancerous tissue (right) and adjacent normal tissue (left). Insets represent enlargements of areas highlighted. Scale bars represent 50 µm. (**C**) Increased Maf1 expression results in repression of RNA polymerase III-dependent transcription. Immunoblot analysis of protein lysates from Huh7 stable cell lines with vector or human Maf1-HA expression plasmid using HA (ectopic Maf1-HA), Maf1, or β-actin antibodies (left). qRT-PCR was performed using RNA isolated from stable cell lines with primers for pre-tRNA^Leu^, pre-tRNA_i_^Met^, 7SL RNA, using GAPDH as an internal control (right). Values are the mean ±S.E. (n≥3). Fold changes in transcripts were statistically different from vector controls (Student t-test, pre-tRNA^Leu^ and pre-tRNA_i_^Met^, *p* = 0.0001, 7SL RNA, *p* = 0.0025). (**D**) Effect of increased Maf1 expression on Huh7 cell doubling time. Stable cell lines described in “C” grown on duplicate plates were trypsinized and counted daily. Values are the mean ±S.E. (n≥3), *p* = 0.0001. (**E**) Increased Maf1 expression represses anchorage-independent growth. Stable cell lines were analyzed for growth in soft agar. Colonies>100 uM were counted. Values are the means ±S.E. (n≥3), *p* = 0.005. (**F**) Tumor growth rate is repressed and visible tumor formation is delayed in mice with cells expressing increased Maf1. Two independent stable cell lines expressing Maf1 were injected subcutaneously into the groins of nude mice (10 mice per group). Calculated tumor growth rates shown (left). The day of first visible tumors noted are shown in the table (right). Values shown are the means ±S.E., *p* = 0.02.

Given that liver and prostate tissues from *Pten* conditional mutant mice exhibit reductions in Maf1 expression, we tested whether Maf1 expression might be similarly deregulated in PTEN-deficient human cancers. Three matched normal tissues and hepatocellular carcinomas that were PTEN negative were examined by immunostaining for potential changes in Maf1 expression. These tissues were also hepatitis B and C negative. A representative case is shown (Fig. 2A). Maf1 was predominantly localized in the nuclei of normal liver tissue. In contrast, matched tumor tissue displayed a marked reduction in nuclear Maf1 expression. We further examined whether Maf1 expression might be deregulated in human prostate cancer where PTEN is frequently lost. Tissue specimens from four human prostate cancer cases that did not express PTEN were examined by immunostaining. A representative case is shown (Fig. 2B). Compared with the predominant nuclear staining of Maf1 in normal prostate epithelium, the adjacent cancerous tissue from the same individual exhibited a substantial decrease in Maf1 expression, correlated with loss of PTEN in the malignant tissue. Together, these results are the first to suggest the idea that Maf1 expression may be deregulated in human cancer. Given that a reduction in Maf1 expression is similarly observed in *Pten*-null mice prostate and liver (Fig. 1A), this suggests that the loss of PTEN drives the alterations in Maf1 expression.

To further ascertain how alterations in Maf1 expression might affect the oncogenic state of cells, stable hepatoma Huh-7 cell lines were created to overexpress Maf1. Ectopic expression of the HA-tagged Maf1 did not alter endogenous Maf1 protein expression. As we could not detect the ectopically expressed higher molecular weight bands of the Maf1-HA protein with Maf1 antibodies, it likely represents a small fraction of the total cellular Maf1 protein. However, this enhanced Maf1 expression resulted in a decrease in Maf1 target gene expression (Fig. 2C). The doubling time of these cells was modestly increased compared with the vector control cells, whereas increased Maf1 expression resulted in a marked reduction in the ability of the cells to form colonies in soft agar (Fig. 2D and 2E). Tumorigenecity assays revealed that while all mice formed tumors, the onset of visible tumors as well as tumor growth was significantly delayed with increased Maf1 expression (Fig. 2F). Together these results reveal that Maf1 represses oncogenic transformation.

### PTEN-mediated regulation of Maf1 occurs via modulation of PI3K/AKT/FoxO1 signaling

**Figure 3:**
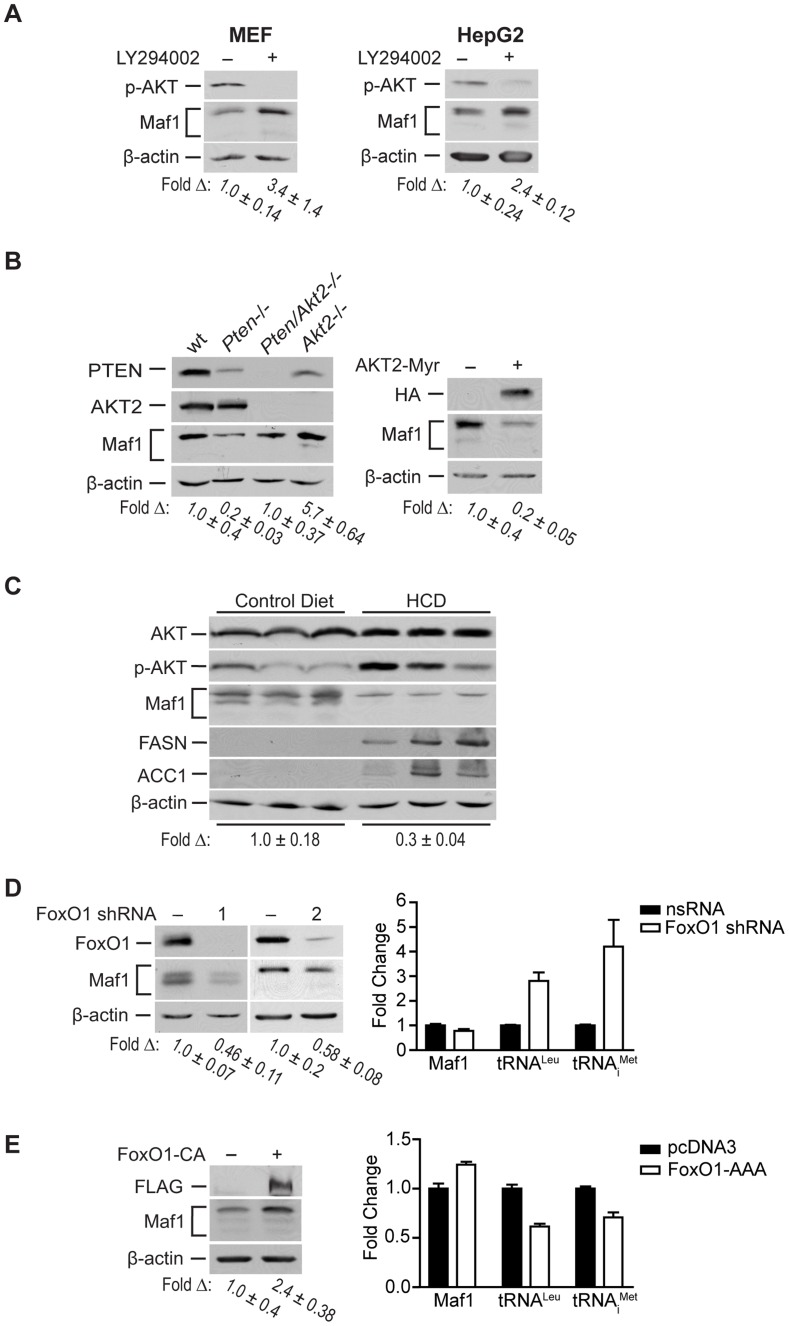
Maf1 is regulated through PI3K/AKT/FOXO1 signaling. (**A**) Pharmacologic inhibition of PI3K signaling increases Maf1 expression. MEF and HepG2 cells were treated with LY294002 or DMSO control for 6 hrs. Protein lysates were subjected to immunoblot analysis. The fold-change in Maf1 was calculated by normalizing to β-actin where the control is set to 1. Values shown are the means ±S.E. (**B**) AKT2 negatively regulates Maf1 expression. Left: Protein lysates from livers of 1 month-old wild-type (n = 4), *Pten*−/− (*Pten^loxP/loxP^; Alb-Cre+*; n = 4), *Pten*−/−; *Akt2*−/− (*Pten^loxP/loxP^; Akt2 null; Alb-Cre+*; n = 3), and *Akt2*−*/*− (*Pten^loxP/loxP^; Akt2 null; Alb-Cre*−; n = 2) mice were subjected to immunoblot analysis. A representative example is shown for each genotype. Right: Huh7 cells were transfected with HA-tagged AKT2-Myr or empty vector control. Protein lysates were subjected to immunoblot analysis with antibodies indicted. The fold-change in Maf1 was calculated by normalizing to β-actin where the control is set to 1. Values shown are the means +S.E. (**C**) Mice fed a high carbohydrate diet display a reduction in Maf1 protein in the liver. Mice were fed control or high carbohydrate diets (HCD), and immunoblot analysis was performed from liver lysates with antibodies against the proteins designated (n = 8 total for each dietary group). The data shown is representative from two independent experiments. The fold-change in Maf1 was calculated by normalizing to β-actin where the control is set to 1. Values shown are the means ±S.E. (**D**) FoxO1 knockdown decreases Maf1 protein expression and increases Maf1 target gene activity. Left: Protein lysates were isolated from MEF cells stably expressing nonsilencing small hairpin RNA (nsRNA) or two distinct FoxO1-targeting shRNAs and immunoblots were performed. The fold-change in Maf1 was calculated by normalizing to β-actin where the control is set to 1. Values shown are the means ±S.E. Right: RNA was isolated from stable MEF cell lines and qRT-PCR was performed with primers specific for precursor tRNA^Leu^ and tRNA_i_^Met^. Values shown are the means ±S.E (n = 3). Values are statistically significant: Student t-test, Maf1, *p = *0.0429; pre-tRNA^Leu^, *p = *0.0001; pre-tRNA_i_^Met^, *p* = 0.011. (**E**) FoxO1 activation positively regulates Maf1 protein expression and represses Maf1 target gene activity. U87 cells were transfected with a FLAG-tagged constitutively active FoxO1 mutant or empty vector control. Protein lysates and RNA were isolated after 48 hrs and subjected to immunoblot analysis (right) and qRT-PCR (left). The fold-change in Maf1 protein levels was calculated by normalizing to β-actin where the control is set to 1. Values shown are the means ±S.E (n = 3). qRT-PCR statistics: Maf1, *p = *0.0029; pre-tRNA^Leu^, *p = *0.0006; pre-tRNA_i_^Met^, *p = *0.0141.

A major function of PTEN is to repress the activation of PI3K signaling. We therefore assessed the potential role of PI3K in regulating Maf1 expression. MEFs and HepG2 human hepatoma cell lines were treated with the PI3K inhibitor, LY294002 (Fig. 3A). Increased Maf1 expression was observed with a corresponding decrease in the activation of AKT. As AKT2 is the predominant form of AKT in liver, we further analyzed its role in regulating Maf1 expression. Maf1 protein expression was measured in lysates derived from wild type livers, and those conditionally deleted for *Pten*, those null for *Akt*, or both (Fig. 3B). Loss of *Akt2* resulted in an increase in Maf1 expression compared with liver lysates derived from wild type mice. Compared with *Pten*-deficient livers, additional loss of *Akt2* restored Maf1 amounts to that observed in wild type mice, supporting a role for AKT2 in negatively regulating cellular Maf1 concentrations. Consistent with these results, expression of a constitutively activated form of AKT2 resulted in a reduction in Maf1 expression in Huh 7 cells (Fig. 3B). Together these results demonstrate that the ability of PTEN to negatively regulate PI3K/AKT signaling is responsible for PTEN-mediated regulation of Maf1 expression.

To determine the biological relevance of Maf1-mediated regulation by PI3K signaling in vivo, we examined how diet-induced activation of this pathway would affect Maf1 expression in mice livers. Mice were fed a high carbohydrate diet (HCD) for 2 days and liver lysates were analyzed by immunoblot analysis. This diet results in insulin-induced activation of *de novo* lipogenesis in the liver [16]. Consistent with these previous results, we observed enhanced phosphorylation of AKT and induction of FASN and ACC1, two limiting enzymes in fatty acid biosynthesis (Fig. 3C). HCD-induced activation of PI3K signaling resulted in a corresponding decrease in Maf1 expression. Together, these results support the idea that Maf1 expression is negatively regulated both *in vitro* and *in vivo* through activation of PI3K signaling.

Because FoxO1 is a direct downstream target of AKT, we sought to determine its role in regulating Maf1. AKT directly phosphorylates and inactivates FoxO1 by causing it to be translocated from the nucleus to the cytoplasm. shRNA-mediated down regulation of FoxO1 resulted in a decrease in Maf1 expression (Fig. 3D, left). Consistent with these results, expression of a constitutively nuclear-persistent and activated form of FoxO1, mimicking AKT inactivation, resulted in enhanced Maf1 expression (Fig. 3E, left). We further assessed how FoxO1 would affect the activity of Maf1-targeted RNA polymerase III-dependent genes. Decreased FoxO1 expression resulted in increases in tRNA gene transcripts (Fig. 3D, right) whereas the expression of the constitutively activated FoxO1 mutant produced a decrease in these transcripts (Fig. 3E, right). Analysis of Maf1 mRNA, however, showed that altered FoxO1 expression or activation produced a relatively modest change in Maf1 mRNA compared with an approximate 2.5 fold change in Maf1 protein. This suggests that FoxO1-induced changes in Maf1 protein are not mediated through the ability of FoxO1 to regulate Maf1 transcription. Together our results reveal that Maf1 expression is positively regulated by FoxO1 and negatively regulated through PI3K/AKT inactivation of FoxO1. In addition, these results identify a new FoxO1-dependent signaling pathway that controls RNA pol III-dependent transcription.

### Maf1 negatively regulates lipogenic gene expression

**Figure 4:**
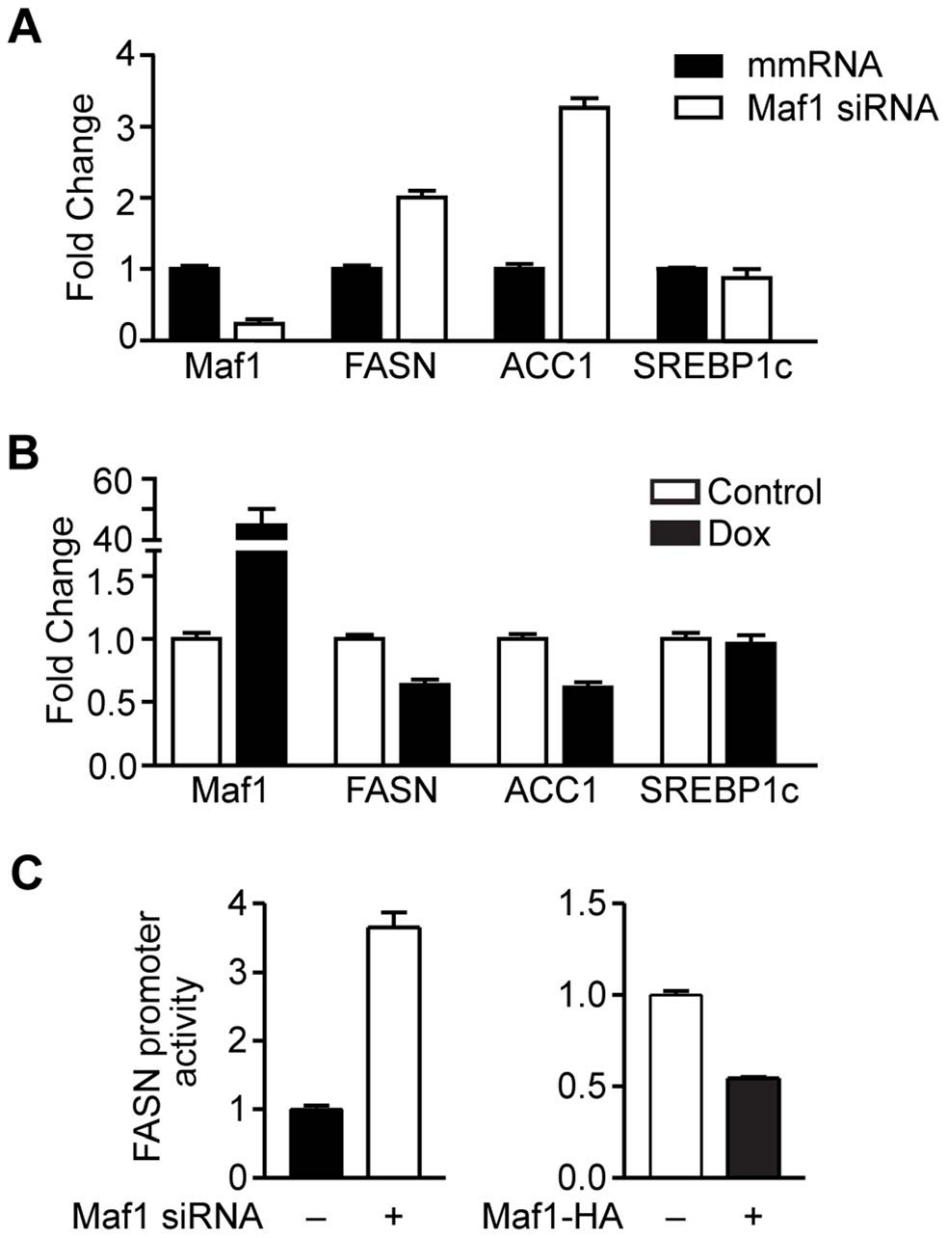
Maf1 negatively regulates fatty acid synthase (FASN) and acetyl-coA carboxylase (ACC1) expression. (**A**) Decreased Maf1 expression increases FASN and ACC1 mRNA. A murine hepatocyte cell line was transiently transfected with non-silencing scrambled mismatch (mm) RNA or siRNA targeting Maf1. RNA was isolated and qRT-PCR was performed. mRNA amounts were normalized to that of GAPDH. (**B**) Increased Maf1 expression represses FASN and ACC1 expression. HepG2 cells were stably infected with Maf1-HA under the control of a doxycycline-inducible promoter. After treatment with 250 ng/ml doxycycline for 48 hrs, RNA was isolated and qRT-PCR was performed. Fold changes are statistically significant with *p*<0.0001. (**C**) Maf1 negatively regulates FASN promoter activity. A murine hepatocyte cell line were transfected with Maf1 siRNA or Maf1-HA and a FASN promoter-reporter construct. Luciferase activity was measured from resulting lysates and normalized to protein levels. Values shown are the means +S.E (n = 4). Fold changes are statistically significant with *p* = 0.0003 (Maf1 siRNA) and *p*<0.0001 (Maf1-HA).

Given the important role of FoxO1 in regulating lipid metabolism [17], and our results indicating that a reduction of Maf1 expression was correlated with an increase in lipogenic enzyme expression in mice fed a high carbohydrate diet, we examined whether changes in Maf1 expression could modulate the expression of lipogenic enzymes. Maf1 expression was decreased in a mouse hepatocyte cell line (Fig. 4A) and we measured the expression of SREBP1c and the lipogenic enzymes, FASN and ACC1. Reduction in Maf1 expression resulted in the enhanced expression of both FASN and ACC1 mRNAs. In contrast, expression of SREBP1c remained unchanged. Ectopic expression of Maf1 in HepG2 cells resulted in a decrease in both FASN and ACC1 expression, but not SREBP1c (Fig. 4B). To determine whether changes in Maf1 expression would alter FASN promoter activity, we used a FASN promoter-reporter construct containing 178 bp upstream of the transcription start site. We observed an induction of the FAS promoter in association with decreased Maf1 expression, whereas ectopic expression of Maf1 resulted in a decrease in FAS promoter activity (Fig. 4C). These results reveal that Maf1 represses the expression of enzymes required for *de novo* lipogenesis. Furthermore, the Maf1-responsive region is contained within the first 178 bp upstream of the transcription start site.

**Figure 5:**
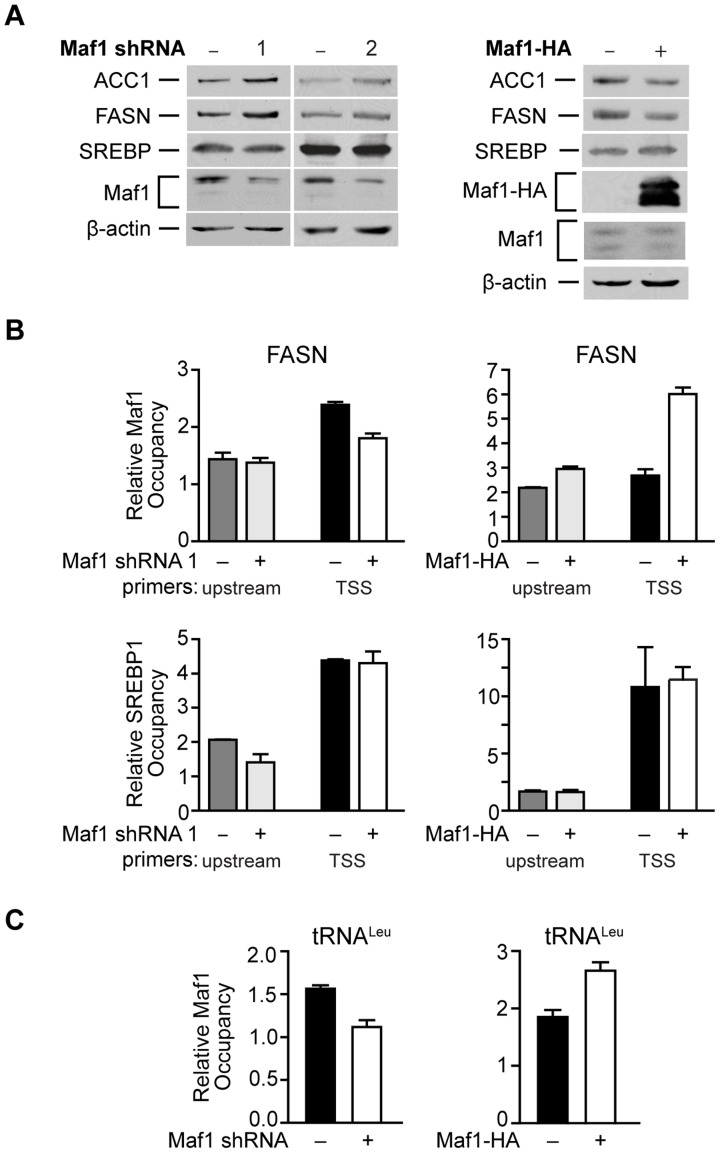
Maf1 occupies the FASN promoter to repress lipogenic gene expression. (**A**) Maf1 negatively regulates cellular FASN and ACC1 protein expression. Protein lysates isolated from Huh7 cells stably infected with nsRNA or Maf1 shRNAs (left) or HepG2 cells engineered to express doxycycline-inducible Maf1-HA as described in Fig. 4 (right) were subjected to immunoblot analysis using antibodies against ACC1, FASN, SREBP, Maf1, HA (right, ectopic Maf1-HA), or β-actin. (**B**) Altering Maf1 levels changes occupancy of Maf1 but not SREBP on the FASN promoter. Chromatin immunoprecipitations were performed with Huh7 cells stably expressing Maf1 shRNA or HepG2-doxycycline-inducible Maf1-HA cells. ChIP analysis was performed with antibodies against Maf1, SREBP1c, and IgG. qPCR was performed with an upstream primers set (gray bars) and a set encompassing the transcription start site TSS (black and white bars). Bars represent Maf1 (top) or SREBP1c (bottom) occupancy relative to input and IgG. Maf1 occupancy at the TSS displayed statistically significant differences, Maf1 siRNA compared to non-silencing control, *p* = 0.001, and doxycycline versus no doxycycline treatment, *p* = 0.0001. (**C**) Altering Maf1 levels changes Maf1 occupancy at the tRNA^Leu^ gene promoter. ChIP analysis was performed as in (B) with primers specific for the tRNA^Leu^ promoter. Values shown for all graphs are the means ±S.E. (n = 4); Student t-test, Maf1 shRNA *p* = 0.0026, Maf1-HA *p* = 0.0013.

We next determined whether Maf1 repression of FASN transcription was mediated by the direct recruitment of Maf1 to the FASN promoter. Consistent with Maf1-mediated changes in mRNAs above, we found that down regulation of Maf1 in Huh7 cells resulted in an increase in both FASN and ACC1 protein expression, whereas ectopic expression of Maf1 in HepG2 cells produced a decrease in these proteins (Fig. 5A). Using chromatin immunoprecipitation assays we asked whether Maf1 was enriched in the region that conferred Maf1-mediated repression in comparison with Maf1 occupancy at sequences upstream of this region. Maf1 occupancy was enriched in sequences encompassing the transcription start site and SREBP1c binding site of the FASN promoter in both Huh7 and HepG2 cells (Fig. 5B, top). We next manipulated Maf1 expression in these cells. Analysis of Maf1 occupancy revealed that down regulation of Maf1 resulted in diminished recruitment of Maf1 to the FASN promoter (Fig. 5B, top left), whereas induction of Maf1-HA expression resulted in the enhanced recruitment of the HA-tagged Maf1 to the FASN promoter (Fig. 5B, top right). These changes in Maf1 occupancy on the FASN promoter observed when cellular Maf1 levels are altered further validate the ChIP signals and were similar to those observed for the Maf1-targeted tRNA^Leu^ gene (Fig. 5C). As expression of SREBP1c protein was unchanged despite altered Maf1 expression (Fig. 5A), we further assessed whether changes in Maf1 expression and promoter occupancy would affect SREBP1c binding. Analysis revealed that changes in Maf1 occupancy did not alter SREBP1c binding to the FASN promoter (Fig. 5B, bottom). These results indicate that at least one mechanism by which Maf1 represses lipogenic gene expression is through the ability of Maf1 to directly target the promoters of these genes. However, given that our previous work revealed that Maf1 represses the expression of the TATA-binding protein, TBP [9], we further assessed whether Maf1-mediated changes in TBP could indirectly regulate lipogenic gene expression. Cellular TBP amounts were increased in Huh7 cells or down-regulated in HepG2 cells (Supplemental Data, Figure S1). In both cases, altered expression of TBP had no affect on the expression of either FASN or ACC1 mRNA. Together, these results support the idea that Maf1 directly targets and negatively regulates genes required for *de novo* lipogenesis and that this effect does not impair the binding of SREBP1c at the promoter.

### Maf1 represses intracellular lipid accumulation and de novo lipogenesis

**Figure 6:**
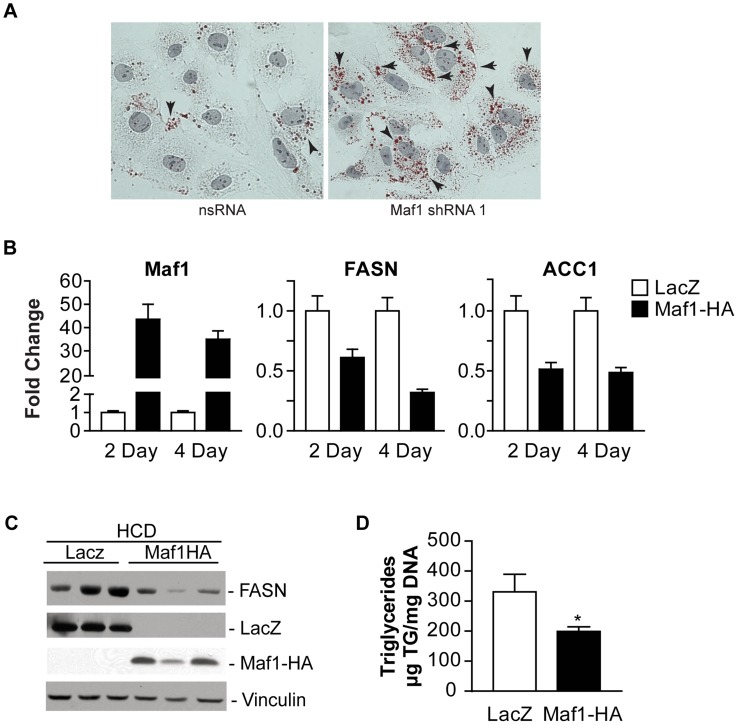
Maf1 controls intracellular lipid accumulation and de novo lipogenesis. (**A**) Maf1 represses intracellular lipid accumulation. Huh-7 cells infected with nsRNA (left) or Maf1 shRNA (right) were stained with Oil Red O and Mayer's Hematoxylin. Intracellular lipid droplets were detected as red spheres and nuclei are shown in purple. Magnification 40x. (**B**) Maf1 overexpression inhibits diet-induced lipogenic gene expression. Male C57BL mice injected with adenovirus overexpressing either Maf1-HA or LacZ were fed a high carbohydrate diet for 2 or 4 days. RNA was isolated from livers and qRT-PCR was performed with primers specific for FASN and ACC1. Fold changes are statistically significant: Student t-test, FASN; 2 day *p* = 0.0067, 4 day *p* = 0.0028; ACC1; 2 day *p* = 0.006, 4 day *p* = 0.021. (**C**) Protein lysates were isolated from the mouse livers fed a high carbohydrate diet for 4 days and were subjected to immunoblot analysis using antibodies against FASN, LacZ, HA (ectopic Maf1-HA), or vinculin. Representative samples shown. (**D**) Maf1 overexpression reduces triglyceride accumulation. Triglycerides were isolated from the livers of mice that were fed a high carbohydrate diet for 4 days and quantified. Values represent means ±S.E. (n = 4). Fold changes are statistically significant with *p*<0.018.

Since the amount of lipogenic enzyme activity controls *de novo* lipogenesis and intracellular lipid accumulation, and FASN expression is limiting for lipogenesis, we further examined the biological consequence of Maf1-mediated repression on lipid biogenesis. As decreased Maf1 expression in Huh-7 cells resulted in an increase in both FASN and ACC1 protein expression (Fig. 5A), we asked whether Maf1-mediated changes in these lipogenic enzymes would affect intracellular lipid accumulation. Compared with control cells expressing non-silencing RNA, cells expressing Maf1 shRNA displayed an increase in the number of visible lipid droplets (Fig. 6A). These results support the idea that Maf1 negatively regulates intracellular lipid accumulation, at least in part, through its ability to directly repress the transcription of lipogenic enzymes.

To further determine whether Maf1 can regulate *de novo* lipogenesis *in vivo*, Maf1 was ectopically overexpressed in mouse livers through adenoviral-mediated gene delivery. Mice were injected into the tail vein with adenovirus expressing an expression vector for HA-tagged human Maf1 or LacZ. Two days following adenovirus delivery, the mice were placed on a high carbohydrate diet for either 2 or 4 days and the livers of the mice were analyzed for RNA and protein expression. Expression of either LacZ or Maf1-HA protein was detected (Fig. 6C). In mice fed a HCD, both Maf1 mRNA (Fig. 6B) and Maf1 protein (Fig. 6C) was significantly increased in the mice injected with the adeno-Maf1-HA compared with the control LacZ construct. The enhanced expression of Maf1 in the liver resulted in a reduction in lipogenic enzyme mRNAs (Fig. 6B) and FASN protein (Fig. 6C) expression. Consistent with these results, there was a significant decrease in liver triglycerides when Maf1 was overexpressed (Fig 6D). Together these results reveal that overexpression of Maf1 in mouse liver is able to repress the expression of the lipogenic enzymes and reduce intracellular lipid accumulation.

## Discussion

Previous studies demonstrated that PTEN negatively regulates RNA pol III-dependent gene transcription through alterations in the phosphorylation state of the transcription factor IIIB components [18]. Our current studies define an additional mechanism by which PTEN represses this class of genes through its ability to regulate Maf1 expression. Our studies identify Maf1 as a novel PTEN target and provide the first evidence that Maf1 functions as a tumor suppressor. Mammalian Maf1 has been shown to repress the expression of both RNA pol III-dependent genes and certain RNA pol II-dependent genes that drive oncogenic transformation [12], [13], [19]. Decreased expression of Maf1 could therefore result in the induction of these genes to promote cellular transformation. Consistent with this idea, enhanced expression of Maf1 significantly represses both anchorage-independent growth and tumor formation in mice. Thus, it is likely that the decrease in Maf1 expression observed in *Pten*-deficient mouse models and human cancer, and the concordant induction of Maf1 gene targets, contributes to the development or progression of the disease. Since deregulation of PTEN is one of the most common aberrations in many different human cancers, and our results show that PTEN-mediated regulation of Maf1 is not cell-type specific, a reduction in Maf1 expression is likely to be observed in many other types of cancers that possess alterations in PTEN function or mutations that activate PI3K signaling. However, it is conceivable that other pathways and mechanisms contribute to deregulation of Maf11 expression or function in human cancers. Collectively, these results support the idea that Maf1 is a key downstream target of PTEN that contributes to the ability of PTEN to function as a tumor suppressor.

Our current studies identify an important new role for Maf1 in regulating PTEN's metabolic functions through its ability to control intracellular lipid accumulation. PTEN-mediated affects on PI3K signaling regulate Maf1 expression to control lipogenic gene expression. Manipulation of Maf1 expression alters intracellular lipids *in vitro*. In vivo, overexpression of Maf1 in mouse liver is capable of abrogating insulin-induced *de novo* lipogenesis. The function of PTEN in repressing lipid metabolism has been well established. In mouse liver, PTEN loss leads to an increase in intracellular lipid accumulation, predominantly through the induction of lipogenesis [14]. In non-alcoholic fatty liver disease (NAFLD), *de novo* lipogenesis plays a significant role in its pathogenesis [20]. Down-regulation of PTEN in NAFLD contributes to steatosis and its progression towards fibrosis and hepatocellular carcinoma [21]. In addition, PTEN expression is repressed in steatotic livers of obese human subjects [22]. Furthermore, the fact that Maf1 is capable of regulating intracellular lipid concentrations in cells and mouse liver that express functional PTEN supports the idea that Maf1 functions downstream of PTEN in this process. As the cellular levels of PTEN are crucial for maintaining homeostasis, aberrant regulation of PTEN, and the subsequent changes in Maf1, may contribute to a variety of disease processes. Consistent with the importance of this newly identified role for mammalian Maf1 in regulating intracellular lipids, *C. elegans* MAFR-1 plays a central physiological function in organismal lipid homeostasis (Khanna et al., accompanying manuscript). Consistent with our findings, the insulin signaling pathway and *daf-18/PTEN* are required for MAFR-1 to regulate *de novo* lipogenesis. Collectively, our results establish a new and conserved Maf1-dependent regulatory node in the maintenance of lipid homeostasis.

Our studies have uncovered a novel mechanism by which downstream PTEN targets, AKT2 and FoxO1, control lipogenic gene expression through Maf1. Our results further identify FoxO1 as a novel critical downstream target of PI3K signaling that controls Maf1 expression to modulate RNA pol III-dependent gene expression, identifying a new signaling pathway that controls both Maf1 expression and the biosynthetic capacity of cells. While Foxo1 is a transcription factor, the very modest effect on Maf1 mRNA relative to the protein changes observed when Foxo1 is manipulated suggests that Foxo1 is not functioning to regulate Maf1 at the level of RNA. There are various ways that Foxo1 might indirectly regulate Maf1 protein expression. FoxO1 could regulate the expression of proteins(s) that associate with Maf1 and/or modify it to regulate its stability. FoxO1 could potentially regulate Maf1 protein synthesis via its ability to regulate the expression of components that enhance the translation of Maf1 mRNA. Additionally, translational regulation could occur through FoxO1 negatively controlling the synthesis of micro RNAs that inhibit Maf1 translation. These various possibilities are currently under investigation.

The physiological role of FoxO1 in regulating hepatic lipid metabolism has been unclear. Deletion of *FoxO1* in mouse liver results in steatosis while *Akt2* deletion is protective for intracellular lipid accumulation that is induced by diet [23] or *Pten* loss [24]. However, concomitant deletion of both *Akt2* and *FoxO1* results in a phenotype similar to the *Akt2* deletion alone [25]. Ectopic expression of constitutively active FoxO1 in *Pten*-deficient mouse liver led to repression of lipogenic gene expression [24]. In addition, activation of mTOR and SREBP1c, a transcription factor that coordinates the induction of the lipogenic enzymes, is not sufficient to drive diet-induced lipogenesis without AKT2 [26]. These results suggest that when insulin signals are not present, alternative pathways may regulate basal lipogenesis independent of forkhead transcriptional factors and SREBP1c. The identification of Maf1 as a novel negative regulator of *de novo* lipogenesis may represent such a signal. Furthermore, AKT2 may control Maf1 expression and function to regulate hepatic lipid metabolism via additional downstream targets.

**Figure 7:**
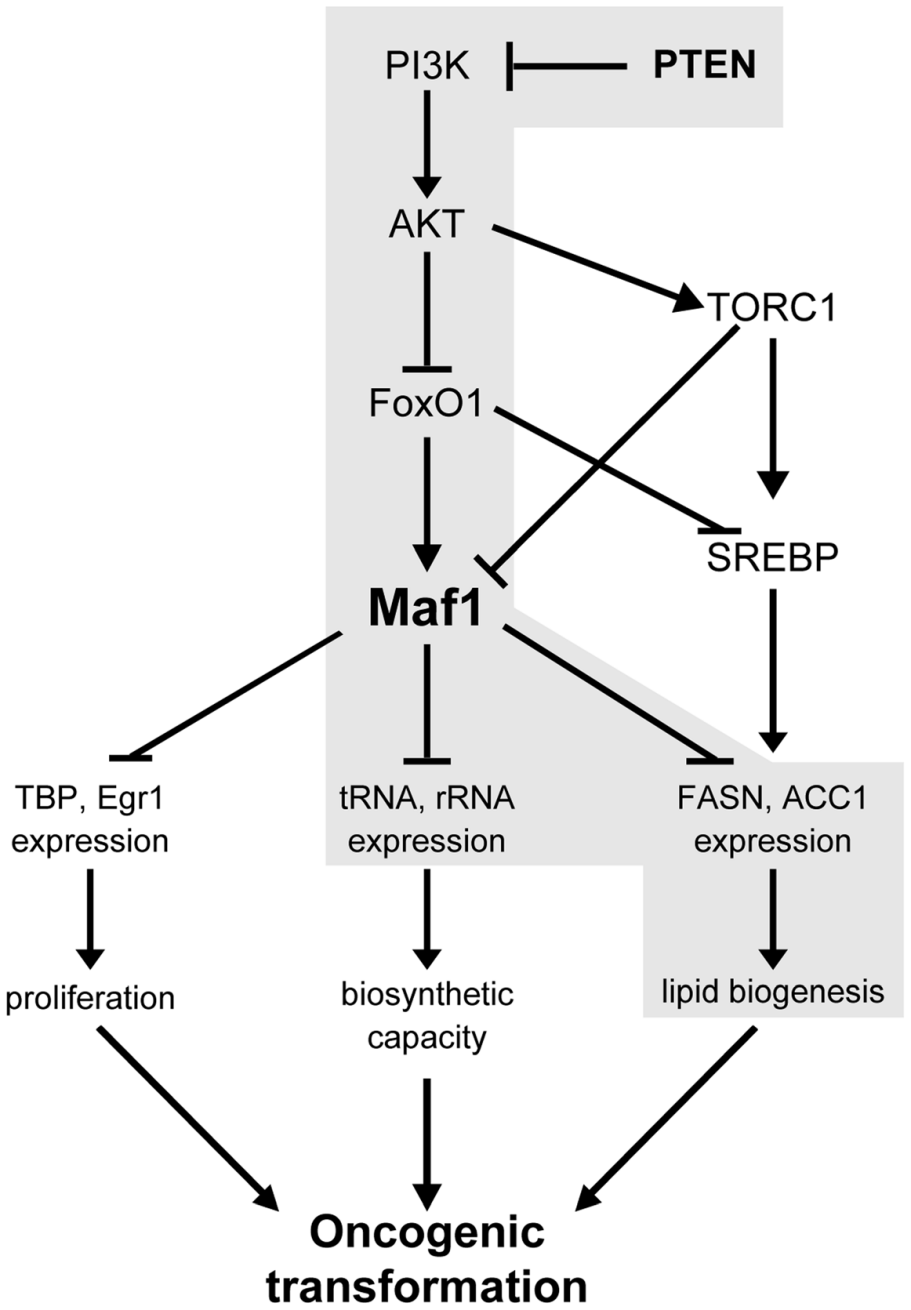
Maf1 is a central coordinator of metabolic signals. Shown is a model depicting the proposed mechanism for the regulation of Maf1 expression by PTEN and the consequences of altered Maf1 expression on gene expression pathways that regulate the oncogenic potential of cells. New findings are highlighted in grey.

Previous work established that PI3K/AKT activation stimulates *de novo* lipogenesis by inducing both the expression and processing of SREBP1c. One downstream AKT target, mTORC1, induces SREBP1c activation [27], [28] whereas another direct target of AKT, FoxO1, represses SREBP1c expression [29], [30] Our results reveal that in addition to its ability to regulate SREBP1c, FoxO1 regulates Maf1 expression to regulate lipogenic gene expression (see schematic, Fig. 7). Previous studies showed that mTORC1 directly phosphorylates Maf1 to decrease its ability to repress RNA pol III-dependent promoters [31], [32]. Together, these results suggest that both mTORC1 and FoxO1 function to control lipogenesis by coordinately regulating both SREBP1c and Maf1. In normal hepatocytes, low nuclear SREBP1c expression and high Maf1 expression and function are likely important for maintaining repression of lipogenic gene expression. Upon AKT activation, decreased cellular expression and function of Maf1 and enhanced nuclear SREBP1 accumulation would allow de-repression and induction of lipogenic genes. Together, our studies support opposing roles for SREBP1c and Maf1 in regulating lipogenic gene expression and lipogenesis.

In yeast, Maf1 selectively represses genes transcribed by RNA polymerase III through its ability to associate with RNA polymerase III itself [8]. Initial studies in mammalian cells revealed that Maf1 also functions to directly repress select RNA pol II-dependent genes [9]. Here, we identify a new category of genes, those encoding lipogenic enzymes, as Maf1 targets. Maf1 is recruited to the FASN promoter within 200 base pairs upstream of the transcription start site to repress gene activity. This region encompasses a binding site for the master lipogenic gene inducer, SREBP1c. Interestingly, changes in Maf1 occupancy do not affect SREPB1c binding. Even in the presence of SREBP1c at the FASN promoter, recruitment of Maf1 is able to override the ability of SREBP1c to induce gene expression. This is consistent with our results demonstrating that increased Maf1 expression in mouse liver is able to diminish FASN and ACC1 expression. Previous studies revealed that Maf1 is recruited to the TBP promoter also within a region close to the transcription start site [9]. Since the Maf1 responsive regions within the TBP and FASN promoters contain distinct transcription factor binding sites, this suggests that Maf1 may repress RNA pol II-dependent gene expression independent of the composition of activator elements present. Maf1 is not known to have a DNA binding domain; therefore, its recruitment to promoters must occur through its association with other transcription components. While Maf1 genome targets and the mechanism by which Maf1 represses gene expression need to be further examined, it is possible that Maf1-mediated repression of RNA pol II-dependent promoters may involve its ability to repress the function or binding of coactivators or components of the general transcription machinery.

Our findings establish a key molecular connection between *de novo* lipid biogenesis and cellular transformation that occurs through the ability of PI3K/AKT signaling to regulate Maf1 expression, which coordinately regulates lipid and protein biosynthesis. Liver-specific *Pten* deletion in mice causes spontaneous development of fatty liver disease starting by 1 month and HCC by 12 months of age [14], [33], [34]. Furthermore, knock out of *Pten* in the liver of *Akt* null mice delays the onset of both fatty liver and tumor formation supporting a role for AKT2 in this process and the notion that the induction of lipogenesis is needed to drive tumor formation [24], [33]. It is well documented that various tumors and their precursor lesions are re-programmed to undergo a significant induction of endogenous fatty acid biosynthesis independent of the levels of circulating lipids [35]. Overexpression and hyperactivity of lipogenic enzymes, such as ACC1 and FASN, have been shown to be critical in the growth and survival of cancer cells [2], [3]. Given our current findings that Maf1 represses intracellular lipid accumulation and cellular transformation, maintaining cellular Maf1 amounts is likely critical for co-repressing gene expression pathways in order to restrain oncogenic transformation. Collectively, our results support the idea that Maf1 serves as a central node in mammalian cells to co-repress genes involved in proliferative, biosynthetic, and metabolic processes (see schematic, Fig. 7). Future studies will be directed towards understanding how aberrant expression or function of Maf1 contributes to risk for metabolic diseases and cancer.

## Materials and Methods

### Cell lines and reagents

Huh7, HepG2, and MEF cell lines were obtained from ATCC. *Pten* deficient MEF [36], murine hepatocyte cell lines [37], and PTEN and PTEN C124S-inducible U87 cell lines [18] were previously generated. PTEN and PTEN C124S cells were treated with 1 µg/ml doxycycline to induce PTEN expression. HepG2 cells were transfected with F1 transfection reagent (Invitrogen). Phosphoinositide 3-kinase inhibitor LY294002 [38] was purchased from Sigma-Aldrich. Mouse Maf1 siRNA target sequence 5′-TGTGACATCTACAGCTATA-3′ was identified using Dharmacon's siDESIGN Center to NCBI accession number NM_026859. Non-silencing mismatch RNA (mmRNA) sequence is described previously [9]. Maf1shRNA2 and Maf1-HA plasmid [9], FoxO1-AAA [24] and FASN luciferase reporter constructs [39] were described previously. Stable Huh7 cell lines were generated by transfection of a Maf1-HA expression construct followed by selection with 200 µg/ml G418. After 4 weeks, G418-resistant cells were pooled. Multiple independent stable cell lines were generated for each construct and experimentally tested and representative results are shown for each.

### Generation of lentiviral constructs

Non-silencing GIPZ lentiviral shRNAmir control vector and GIPZ lentiviral shRNAmir expression clones to human Maf1 or human TBP were from Thermo Open Biosystems (Maf1: Clone IDs V2LHS_138266, 3′ UTR target; V3LHS_380771, cDNA target; V3LHS_380770, cDNA target; TBP: V2LHS_153794 (TBP shRNA 1), 3′ UTR target, V2LHS_262152 (TBP shRNA2), 3′ UTR target). Non-silencing empty vector control and pLKO.1-mouse FoxO1 shRNA (clone IDs TRCN0000054880 and TRCN0000054879) were from Thermo Open Biosystems.

Maf1-HA from pcDNA 3.1(−) Maf1-HA [9] was PCR amplified to add BamHI and a Kozak consensus sequence 5′ and XbaI 3′ of the Maf1-HA cDNA. E2-TBP from pLTR-E2TBP [11] was PCR amplified to add BamHI 5′ and XbaI 3′ of double HA epitope tagged human TBP. Inducible lentiviral vector pFTREW [40] was restriction digested with BamHI and XbaI to remove GFP and subclone in Maf1-HA (pFTREW-Maf1-HA) or E2TBP (pFTREW-E2TBP). FTREW and FUIPW-rtTA (lentiviral tetracycline transactivator) vectors were a gift from Wange Lu (USC) [40].

Lentiviral particles were produced by transfection of HEK293T using calcium phosphate. Lentiviral vectors were transfected with pMD2.G (vesicular stomatitis virus envelope protein (VSV-G) expression vector) and psPAX2 (packaging vector). After 48 h, conditioned media was collected, sterile filtered and centrifuged at 100,000×*g* (90 min, 4°). Pelleted virus was resuspended in media and sterile filtered. Cell lines were transduced with concentrated virus from conditioned media for 6–16 h. After 3 days, the infected cells were selected for with puromycin or GFP expression determined by fluorescence microscopy.

### Adenoviral-mediated gene delivery

Adenovirus (type 5 dE1/E3) overexpressing human Maf1-HA or LacZ cDNAs under control of the CMV promoter were purchased from Vector Biosystems. Virus diluted in PBS (1×10^9^ plaque-forming units (pfu)) was injected into the tail vein of 6–8 weeks old C57/BL6 male mice. Animals on a standard chow or high carbohydrate diet were sacrificed 4 and 6 days post injection and the livers were perfused with PBS and isolated. Three independent experiments were conducted with a minimum of 5 mice per group.

### Cell accumulation rates, anchorage independent growth, and athymic mouse tumorigenicity assays

Accumulation rates of Huh7 cells stably transfected with Maf1-HA or vector control expression plasmid were determined by plating 50,000 cells per 60 mm dish. Cells were harvested at daily and viable cells were counted using a hemacytometer and trypan blue exclusion. Growth in soft agar assays and tumorigenicity assays with athymic nude (nu/nu) mice were conducted as previously described [11]. Briefly, for the tumorigenecity assays, stable Huh7-Maf1-HA or vector control cells were resuspended in 1∶1 media:matrigel and 2×10^6^ cells/100 µl injected per animal. Tumor volume was measured twice weekly. The first day for visible tumor was determined based when two consecutive tumor volume measurements increased 50% or more. The rate of tumor growth (tumor volume/day) was determined by linear regression on the tumor growth curves in log phase growth to measure the slope (R^2^>0.8). Statistics to determine significant differences (*p*<0.05) were performed using the Student's T-test.

### Mouse tissues

*Pten^loxP/loxP^*; *Alb-Cre^+^*, and *Pten^loxP/loxP^*; *Alb-Cre^+^*; *Akt2*−*/*− double mutant mice were previously generated [14], [24]. Livers were harvested, flash frozen in liquid nitrogen, and prepared for protein analysis as previously described [14]. *Pten^loxP/loxP^*; *PB-Cre* ^+^ mice were generated and prostate tissue was processed for protein analysis as previously described [15]. C57BL/6 mice (16 wk old; Jackson Labs) were fed standard chow diet or high carbohydrate diet for 2 days as described previously [41] and livers were harvested for protein analysis. All experiments were performed according to guidelines of the University of Southern California Institutional Animal Care and Use Committee.

### Immunoblot analysis

Protein lysates were subjected to immunoblot analysis as previously described [9] using the following antibodies: Maf1 (Abcam), PTEN, p-AKT, AKT2, FoxO1, ACC1 (Cell Signaling), SREBP-1c (Santa Cruz), HA (Roche), FLAG (Sigma), FASN (BD), TBP (N-12, Santa Cruz), and β-actin (Sigma). Bound primary antibody was visualized using HRP-conjugated secondary antibodies (Pierce) or biotinylated secondary antibodies complexed with avidin/peroxidase (Vector Labs) and enhanced chemiluminescence reagents (Pierce). Densitometry was performed using UN-SCAN-IT software (Silk Scientific).

### Quantitative Real-Time PCR

Total RNA was isolated and reverse transcribed as previously described [9]. RT-qPCR was performed with Brilliant II SYBR Green qPCR Mastermix (Stratagene) on the MX3000P System (Stratagene). Maf1 primer sequences are: (F) 5′-GTG GAG ACT GGA GAT GCC CA-3′; (R) 5′- CTG GGT TAT AGC TGT AGA TGT CAC A-3′ Primer sets for FASN, ACC1, SREBP1c [24], pre-tRNA_i_^Met^ [12], pre-tRNA^Leu^ and GAPDH [9] have been described previously. Relative amounts of transcripts were quantified by the comparative threshold cycle method (ΔΔCt) using GAPDH as the endogenous reference control. Fold change was calculated from the control or vector cell lines.

### Chromatin immunoprecipitation

Chromatin immunoprecipitation was performed as previously described [9]. DNA was amplified by quantitative PCR using Terra qPCR Direct SYBR Premix (Clontech) or Brilliant II SYBR Green qPCR Mastermix (Stratagene) on the MX3000P System (Stratagene). FASN primer sequences are: −2479 to −2279 upstream forward 5′-CTG GTC ACA CTC TGC CCA CAG C-3′, reverse 5′-AGC TGC AAA GGT CCC ACG A-3′, and transcription start site -104 to +132 forward 5′-CAG CCC CGA CGC TCA TTG G-3′, reverse 5′-GGC TGC TCG TAC CTG GTG AG -3′. Primers for the tRNA^Leu^ gene were described previously [9]. Ct values for antibody pulldowns were normalized to input using the antibody IP*10/input calculation and IgG.

### Immunohistochemistry

For intracellular lipid staining, Huh7 stable cell lines were plated on coverslips and grown overnight at 37°. Cells were fixed and stained with Oil Red O and hematoxylin. Human tissues were obtained from the USC/Norris Comprehensive Cancer Center Tissue Procurement Core. Hematoxylin and eosin staining was performed to identify normal and cancerous tissue. Human liver sections and prostate sections were stained with antibodies against Maf1 and PTEN and counterstained with hematoxylin. The Maf1 antibodies (Santa Cruz FL-256) were validated for immunohistochemistry staining by Santa Cruz.

## Supporting Information

Figure S1 Changes in TBP expression do not affect FASN or ACC1 mRNA expression. (**A**) Huh7 cells were stably infected to express a doxycycline-inducible double HA-tagged human TBP cDNA. Cells were induced with 800 ng/ml doxycycline for 16 h and protein and RNA isolated. *Left*: TBP and actin immunoblots. Relative TBP protein amounts was normalized to β-actin and Dox (−) value set to 1. A representative blot is shown. *Right*: qRT-PCR was performed with primers specific for Maf1, FASN and ACC1. (**B**) HepG2 cells were stably infected to express nsRNA or TBP shRNAs. Protein and RNA were isolated. *Left*: TBP and actin immunoblots. The relative amount of TBP was normalized to β-actin and nsRNA (−) value set to 1. A representative blot is shown. *Right*: qRT-PCR was performed with primers specific for Maf1, FASN and ACC1. Three independent experiments were performed for each analysis.
(TIF)
