# Correction: Maf1 Is a Novel Target of PTEN and PI3K Signaling That Negatively Regulates Oncogenesis and Lipid Metabolism

**DOI:** 10.1371/journal.pgen.1005055

**Published:** 2015-03-18

**Authors:** The PLOS Genetics Staff

In the Discussion, Khanna et al. is described as an accompanying manuscript. The correct reference for this paper is: Khanna A, Johnson DL, Curran SP (2014) Physiological Roles for *mafr-1* in Reproduction and Lipid Homeostasis. Cell Rep. 9(6): 2180–2191. doi: 10.1016/j.celrep.2014.11.035.
