# Supplementary figures and images for: Maf1 Is a Novel Target of PTEN and PI3K Signaling That Negatively Regulates Oncogenesis and Lipid Metabolism
Source: PLoS Genet. 2014 Dec 11;10(12):e1004789. doi: 10.1371/journal.pgen.1004789 (PMC4263377; doi:10.1371/journal.pgen.1004789)

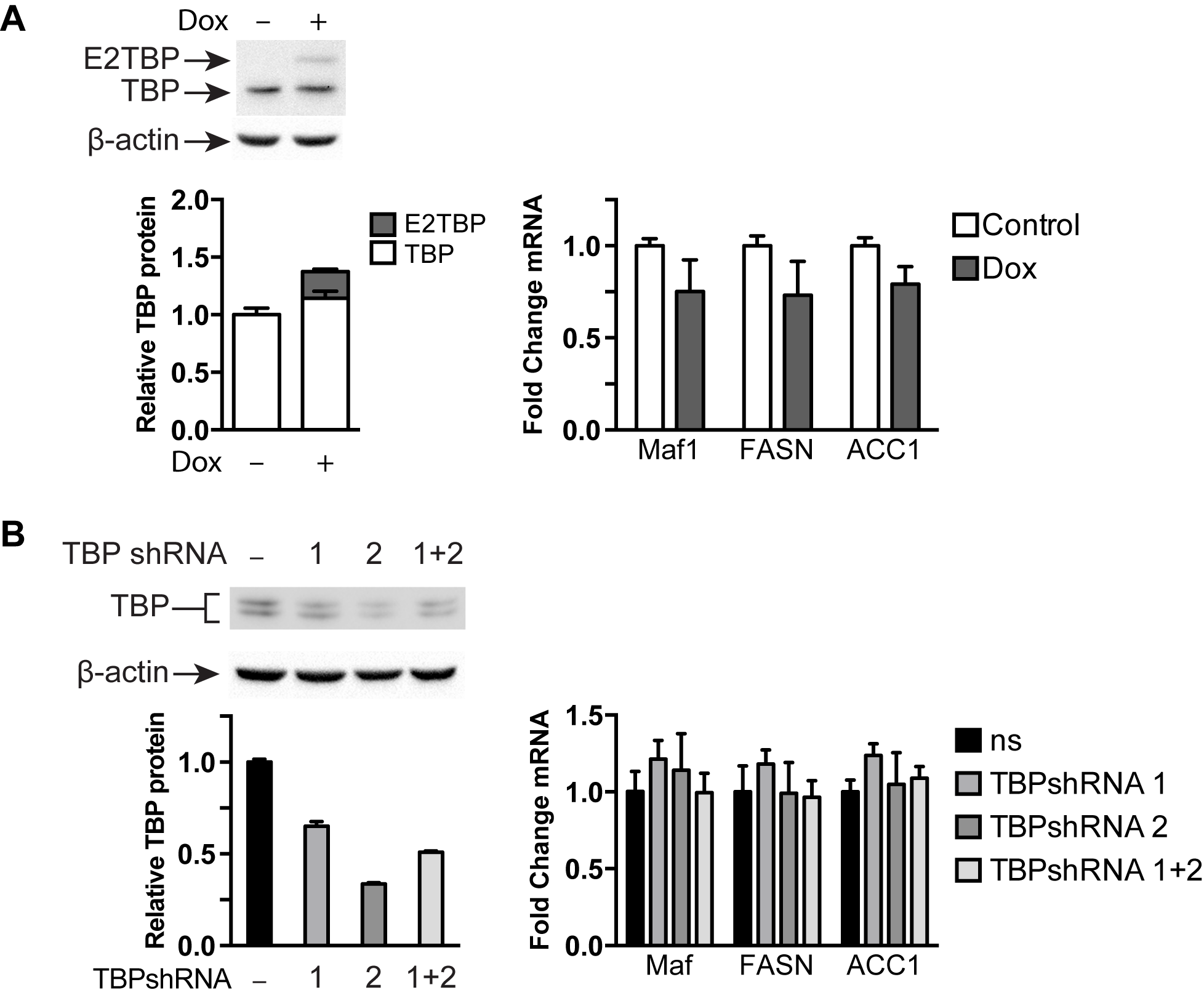

Supplement: Figure S1 — Changes in TBP expression do not affect FASN or ACC1 mRNA expression. (A) Huh7 cells were stably infected to express a doxycycline-inducible double HA-tagged human TBP cDNA. Cells were induced with 800 ng/ml doxycycline for 16 h and protein and RNA isolated. Left: TBP and actin immunoblots. Relative TBP protein amounts was normalized to β-actin and Dox (−) value set to 1. A representative blot is shown. Right: qRT-PCR was performed with primers specific for Maf1, FASN and ACC1. (B) HepG2 cells were stably infected to express nsRNA or TBP shRNAs. Protein and RNA were isolated. Left: TBP and actin immunoblots. The relative amount of TBP was normalized to β-actin and nsRNA (−) value set to 1. A representative blot is shown. Right: qRT-PCR was performed with primers specific for Maf1, FASN and ACC1. Three independent experiments were performed for each analysis. (TIF) [file pgen.1004789.s001.tif]
